# Handgrip strength is associated with mortality in community-dwelling older adults: the Yilan cohort study, Taiwan

**DOI:** 10.1186/s12889-023-17058-9

**Published:** 2023-11-08

**Authors:** Nai-Wei Hsu, Ching-Heng Lin, Nan-Ping Yang, Hsi-Chung Chen, Pesus Chou

**Affiliations:** 1 Community Medicine Research Center & Institute of Public Health, School of Medicine, National Yang Ming Chiao Tung University, Taipei, Taiwan; 2 Division of Cardiology, Department of Internal Medicine, National Yang Ming Chiao Tung University Hospital, Yilan, Taiwan; 3 Public Health Bureau, Yilan County, Yilan, Taiwan; 4 Department of Medical Research, Taichung Veterans General Hospital, Taichung, Taiwan; 5 Department of Orthopedic Surgery, Taoyuan General Hospital, Ministry of Health & Welfare, Taoyuan, Taiwan; 6 Department of Psychiatry & Center of Sleep Disorders, National Taiwan University Hospital, Taipei, Taiwan

**Keywords:** Handgrip strength, Mortality, Community-dwelling older people

## Abstract

**Introduction:**

Hand grip strength (HGS) is one of the methods to help early identification of physical frailty and sarcopenia, the major concerns in the aging societies. It is also crucial to evaluate its impact on mortality. However, the available evidence regarding such impact among specific age cohorts (65 to 74 years and above) is limited. This study tried to investigate the relationship between HGS and mortality among specific cohorts of the community-dwelling older individuals in Yilan, Taiwan.

**Methods:**

A seven-year longitudinal follow-up study was conducted involving 2,468 community-dwelling older individuals in Yilan. The participants were divided into two groups based on their quartiles of hand grip strength: with poor HGS and with good HGS. The association between HGS and mortality was examined using Cox proportional hazards models.

**Results:**

The analysis revealed that age, HGS, gender, medical history of cardiovascular diseases, body mass index, and wrist-hip ratio had significant impacts on seven-year survival. Specifically, individuals with poor HGS exhibited increased mortality, with an adjusted hazard ratio (HR) of 1.87 (95% CI: 1.52–2.30). Furthermore, the adverse effect of poor HGS on mortality was more pronounced in males aged 65–74 years (adjusted HR 4.12, 95% CI: 2.16–7.84), females aged 75 years or older (2.09, 1.43–3.04) and males aged 75 years or older (1.49, 1.07–2.07).

**Conclusion:**

Poor hand grip strength is an independent risk factor for mid-term mortality among community-dwelling older individuals in Yilan. The assessment of HGS can serve as a valuable tool in identifying older individuals at higher risk of death.

**Supplementary Information:**

The online version contains supplementary material available at 10.1186/s12889-023-17058-9.

## Introduction

In societies experiencing rapid aging, the impacts of noncommunicable diseases (NCDs) and subsequent mortality become increasingly significant, particularly in the aftermath of a pandemic infectious disease. However, even with aggressive and huge efforts, Murray et al. reported that the record for reducing exposure to harmful risks, such as high fasting glucose and high body mass index (BMI), of the world over the past three decades was still poor. Besides, high systolic blood pressure with subsequent hypertension has accounted for 10.8 million deaths in 2019 [1]. Therefore among those vulnerable groups, such as the older people, physical frailty [2, 3] and sarcopenia [4, 5] emerge as crucial concerns because of their possible preventability and reversibility. To address this issue, researchers have explored several simple and easily assessed methods, including hand grip strength (HGS), as indicators of physical frailty and sarcopenia. Besides, being one of the components of diagnostic criteria of these syndromes, the impacts of HGS on the health outcomes will also provide more information for further effective health strategies making.

Studies have demonstrated the value of HGS in predicting mortality risk in various contexts, such as cardiovascular disease (CVD) [6–10], cancer [11–13], and hip fracture [14–16]. Besides, inverse associations between HGS and overall mortality have been identified in patients with non-alcoholic fatty liver disease (NAFLD) [17], type-2 diabetes (T2D) and advanced heart failure (HF) [18, 19]. Moreover, a longitudinal study based on the Osteoporotic Fractures in Men (MrOS) USA Study found that each 1-SD annual decrement in the change in HGS was associated with an increased post-fracture mortality, with a HR of 1.15 (95% CI, 1.01–1.33) [16].

In the field of community medicine and preventive medicine, HGS has also emerged as a suitable measurement for large-scale screening of strength and weakness among community-dwelling older individuals. In several large-scale studies, such as the longitudinal surveys conducted in Eastern and Western countries, the relationship between HGS and mortality risk has been investigated [7, 20–23]. Notably, studies based on the China Health and Retirement Longitudinal Study (CHARLS) have revealed that lower HGS is an independent predictor of mortality among middle-aged and older individuals in China [24, 25]. Similarly, the Survey of Health, Ageing and Retirement in Europe (SHARE) has shown an inverse linear dose–response association between objectively measured HGS and all-cause mortality risk in older adults [26, 27]. Besides, several systematic reviews and meta-analyses have also been conducted to assess the association between HGS and mortality risk. Even with various and heterogeneous protocols, these studies consistently supported HGS as a reliable predictor of mortality risk [28–30]. However, Gómez-Campos et al. have demonstrated there was a nonlinear relationship between chronological age with HGS from infancy to senescence, with men experiencing an accelerated and continuous reduction of HGS and women with a smaller reduction of HGS after the age of 40 years [31]. Riviati et al. have also suggested that age over 75 years old alone increased the risk of low HGS in older people [32]. As lower HGS have been shown to be associated with higher mortality risk [33, 34] and the absolute values of HGS of various genders were different [35], it was reasonable to estimate that while we stratified older population into more specific age and gender cohorts, the impacts of HGS on mortality might be distinct. But the available evidence was still limited because the majority of studies did not specifically analyze the age cohorts of 65–74 years and 75 years and above [8–10, 24, 27], and some studies did not separate the analysis by gender as well [7, 10].

The present community-based survey in Taiwan aimed to address this research gap. The study had two primary objectives: (1) to investigate the relationship between HGS and mortality risk and to estimate mortality rates, and (2) to estimate the hazard ratios (HRs) of HGS on mortality of specific age and gender cohorts in the older population. By establishing this link and taking different life styles and comorbidities into consideration in various population cohorts, the study tried to offer valuable insights that could help to design future interventions, esp. aiming at management of physical frailty and sarcopenia in older people in Taiwan, more effective and efficient.

## Methods

### Study design

As part of the Yilan study series, a cluster of studies focused on HGS have been implemented with standardized procedures as well [35–38]. The target population for the study consisted of randomly selected residents aged 65 years or older from Yilan City, located in northeastern Taiwan. The present study duration with HGS measurement spanned from February 2013 to the end of 2016. Participants who expressed willingness to take part in were enrolled, while those who declined were excluded from the study. In the context of the HGS-related studies, individuals who had preexisting health problems, such as previous cerebrovascular disease with limb weakness, various kinds of arthritis, hand trauma or previous hand surgery, with resultant difficulty in hand movements were additionally excluded from the HGS measurement. The study followed the enrolled individuals for a period of 7 years, with data collection concluding in August 2020.

### Data collection

Data collection involved trained project assistants conducting face-to-face interviews with the enrolled participants at their respective homes. After obtaining the participant’s consent, that assistants would ask the older adult to rest for 5 min and proceeded the interview using the designed questionnaire (including basic demographic information, such as age, gender, educational level, life style, and the self-reported medical history). Following that, the HGS of both hands were checked. Finally, the participant’s body weight, height, waist and hip circumstance were also measured.

### Measurement of handgrip strength and mortality status

HGS was measured using a hydraulic hand dynamometer (JAMAR® Hydraulic Hand Dynamometer, Model 5030J1; Performance Health Supply, Inc., USA). During the measurement, we asked the participants to sit in the neutral position with the elbow at their sides flexed at right angles. Because sometimes it was not easy to identify the dominant hands in these older adults, therefore each participant would undergo two trials for each hand at the interval of 15–30 s, and the best performance from each hand was recorded. The average of the best performances was then calculated and utilized as the final estimate of hand grip strength [21, 35–37, 39]. In this study, poor HGS was operationally defined as a grip strength falling below the 25th percentile for a given age and gender. For the 65–74 age cohort, grip strength below 23.5 kg for men and 14.2 kg for women were classified as poor grip strength. For the 75 and older age cohort, grip strength below 16.6 kg for men and 10.0 kg for women were considered indicative of poor grip strength.

Mortality events were identified by referring to death registries. Mortality status was determined based on whether an individual was recorded as having died during the follow-up period. Vital records were utilized as the primary source of information for establishing an individual's mortality status.

### Covariates

In this study, factors that were available in Yilan study and were well-established risk indicators for mortality were specified as potential confounders for the relationship between HGS and mortality risk.

### Measurement of waist and hip circumference

We measured the participants’ waist and hip circumferences according to the guidelines from the Health Promotion Administration of Ministry of Health and Welfare of Taiwan. By asking them to stand in neutral position at first, the project assistants measured the waist circumference at the level just in the middle between the lower margin of the rib and the upper margin of the ilium of the participants at the end of their expiration. We also measured the circumference of the widest part of buttock as their hip circumference. The waist-hip ratio (WHR) was derived from the waist circumference divided by the hip circumference.

### Lifestyles and morbidities

The participants’ education status has been categorized into illiterate, primary school, secondary school and above. Lifestyle factors, including smoking and drinking status, were obtained through self-report. The medical history of chronic diseases, including CVD, hypertension, diabetes, and hyperlipidemia, has been obtained by first asking the participant whether she or he has been diagnosed with such disease. If the answer was yes, she or he would be asked another question: Have you ever received any kind of treatment for such disease? Only those who reported “yes” to these 2 questions were coded as having a “presence” of this specific disease. All others were coded as having an “absence” of this disease.

### Ethical approval

The Yilan cohort study has been conducted by the Community Medicine Research Center of the National Yang Ming Chiao Tung University and the National Yang Ming Chiao Tung University Hospital in Taiwan, with the objective of assessing community health. The study protocol has underwent evaluation and received approval from the Institutional Review Board (IRB) of National Yang Ming Chiao Tung University Hospital (IRB Approval No.: 2011A016, 2014A004, and 2017A014), which holds certification from the Ministry of Health and Welfare, Taiwan. Additionally, the questionnaire utilized in the Yilan study obtained approval from the same IRB. Written informed consent forms were obtained from all enrolled participants, indicating their voluntary agreement to participate in the study. Furthermore, all interviews and measurements were conducted in compliance with the applicable guidelines and regulations in Taiwan.

### Statistics

Descriptive statistics were presented as frequencies and percentages, and the chi-square test was employed to assess significant differences among groups. To conduct multivariable analyses, hazard ratios (HR) with corresponding 95% confidence intervals (CI) were calculated using the Cox proportional regression method. Proportional hazards assumption was tested to ensure the validity of the Cox regression analysis. The assumption was evaluated through graphical methods, such as log–log survival plots and Schoenfeld residuals plots, which showed parallel lines. These findings collectively support the fulfillment of the proportional hazards assumption. In this study, a full model with all covariates forcedly entered the model as potential confounders was specified to examine the independent impact of HGS on mortality risk. In addition, a stepwise method was employed for variable selection with the aim of identifying the most influential predictors or features that significantly contribute to the predictive power of the model. In variable selection, variables with *p*-values < 0.05 were added, while those with *p*-values > 0.10 were considered for removal. This method aimed to balance meaningful predictors' inclusion while preventing less significant ones, reducing overfitting risks. By utilizing the stepwise method, variables were prioritized based on their statistical significance and their impacts on the outcome. This approach enables us to gain insights into the importance of the rank of HGS in relation to mortality. Additionally, adjusted HRs and survival curves based on the Kaplan–Meier estimator were calculated and graphically depicted, specifically stratified by age and gender.

## Results

### Enrolled population

The present study employed a cohort design to investigate the association between HGS and all-cause mortality with a total follow-up period of 7 years. A total of 2,468 participants from the Yilan cohort study was enrolled, consisting of 1,436 females (58.2%) and 1,032 males (41.8%). Among these participants, 609 individuals (24.7%) were classified as having poor HGS, falling below the 25th percentile for their respective age and gender. The remaining 1,859 participants were categorized as having good HGS. Participants were followed up over time to determine the incidence of mortality using death registries. During the average 4.5 years follow-up time, 498 death events were recorded.

### Baseline characteristics

Table 1 presents the baseline characteristics of all the subjects included in the study. The majority of the studied participants were female, accounting for 57.8% and 58.3% in the poor and good HGS groups, respectively. Similarly, a large proportion of participants in both groups were aged 75 years or older, with percentages of 60.1% and 60.5%, respectively. These differences in gender distribution and age between the two groups were not statistically significant. However, the poor HGS group exhibited significantly higher proportions of individuals with an illiterate education level, underweight status, abnormal WHR, and a non-alcohol drinking lifestyle compared to the good HGS group (35.0% vs. 28.0%, 6.6% vs. 3.5%, 60.0% vs. 52.9%, and 86.2% vs. 80.5%; *p* = 0.003, 0.005, 0.004, and 0.001, respectively). Additionally, a significantly higher prevalence of CVD and diabetes, based on medical history, was observed in the poor HGS group compared to the good HGS group (40.6% vs. 31.1%, *p* < 0.001, and 29.1% vs. 23.1%, *p* = 0.003, respectively).

**Table 1:** Characteristics of study subjects by hand grip strength

Variables	Hand grip strength		
	Good	Poor	*P*-value
	(*n* = 1859)	(*n* = 609)	
	n (%)	n (%)	
Gender			0.824
Female	1084 (58.3)	352 (57.8)	
Male	775 (41.7)	257 (42.2)	
Age (years)			0.855
65–74	734 (39.5)	243 (39.9)	
≥ 75	1125 (60.5)	366 (60.1)	
Education level (missing = 4)			0.003
Illiterate	521 (28.0)	212 (35.0)	
Primary school	724 (39.0)	224 (37.0)	
Secondary school and above	613 (33.0)	170 (28.1)	
BMI (kg/m^2^) (missing = 146)			0.005
< 18.5	63 (3.5)	34 (6.6)	
18.5–23.9	699 (38.7)	212 (41.0)	
24–26.9	579 (32.1)	140 (27.1)	
≥ 27	464 (25.7)	131 (25.3)	
Waist-hip ratio (missing = 137)			0.004
Normal	854 (47.1)	207 (40.0)	
Abnormal (> 0.95 for male; > 0.85 for female)	959 (52.9)	311 (60.0)	
Lifestyle			
Smoking			0.517
Never	1380 (74.2)	453 (74.4)	
Current smoker	181 (9.7)	51 (8.4)	
Quit	298 (16.0)	105 (17.2)	
Alcohol drinking			0.001
Never	1496 (80.5)	525 (86.2)	
Current drinker	264 (14.2)	52 (8.5)	
Quit	99 (5.3)	32 (5.3)	
Medical history			
Cardiovascular diseases (missing = 1)	577 (31.1)	247 (40.6)	< 0.001
Hypertension	1085 (58.4)	373 (61.2)	0.209
Diabetes	430 (23.1)	177 (29.1)	0.003
Hyperlipidemia (missing = 26)	421 (22.9)	137 (22.6)	0.890

### Association between handgrip strength and mortality

A multivariable Cox proportional hazard model (full model) was utilized to assess the factors associated with mortality among the community-dwelling older participants, as shown in Table 2. For the overall sample, death was significantly associated with older age (75 years or more), poor HGS status, male gender, a medical history of CVD, underweight (BMI < 18.5), and abnormal WHR. The hazard ratios (HRs) for these factors were 2.67 (95% CI: 2.09–3.41), 1.85 (95% CI: 1.50–2.28), 1.73 (95% CI: 1.30–2.29), 1.66 (95% CI: 1.35–2.03), 1.82 (95% CI: 1.25–2.65), and 1.29 (95% CI: 1.03–1.62), respectively. Furthermore, compared to individuals with normal body weight, those who were overweight (BMI 24–26.9) and obese (BMI ≥ 27) exhibited a lower risk of mortality, with HRs of 0.70 (95% CI: 0.54–0.90) and 0.75 (95% CI: 0.57–0.98), respectively. Furthermore, a stepwise Cox proportional hazard model was conducted, with age, HGS, and gender entered as the first, second, and third variables, respectively. Subsequently, CVD medical history, BMI, and WHR were included. Focusing on HGS status, poor HGS was associated with an increased risk of mortality, with an adjusted HR of 1.87 (95% CI: 1.52–2.30).

**Table 2:** Unadjusted and multivariable Cox proportional hazard models of death

	Unadjusted Models			Full model			Stepwise model			
Variables	Hazard ratio	95%CI	*P*-value	Hazard ratio	95%CI	*P*-value	Step	Hazard ratio	95%CI	*P*-value
Age (years)							1st			
65–74	1.00	–-		1.00	–-			1.00	–-	
≥ 75	3.57	2.85–4.49	< 0.001	2.67	2.09–3.41	< 0.001		2.76	2.17–3.51	< 0.001
Hand grip strength							2nd			
Good	1.00	–-		1.00	–-			1.00	–-	
Poor	2.28	1.91–2.73	< 0.001	1.85	1.50–2.28	< 0.001		1.87	1.52–2.30	< 0.001
Gender							3rd			
Female	1.00	–-		1.00	–-			1.00	–-	
Male	1.87	1.57–2.24	< 0.001	1.73	1.30–2.29	< 0.001		1.96	1.60–2.41	< 0.001
Medical history										
Cardiovascular diseases	1.97	1.66–2.35	< 0.001	1.66	1.35–2.03	< 0.001	4th	1.71	1.40–2.07	< 0.001
Hypertension	1.24	1.03–1.48	0.022	1.13	0.91–1.41	0.273	–-			
Diabetes	1.09	0.90–1.34	0.381	0.98	0.78–1.24	0.879	–-			
Hyperlipidemia	0.87	0.70–1.08	0.210	0.93	0.72–1.19	0.566	–-			
BMI (kg/m^2^)							5th			
< 18.5	2.09	1.48–2.95	< 0.001	1.82	1.25–2.65	0.002		1.78	1.23–2.58	0.002
18.5–23.9	1.00	–-		1.00	–-			1.00	–-	
24–26.9	0.67	0.53–0.85	< 0.001	0.70	0.54–0.90	0.005		0.69	0.54–0.89	0.004
≥ 27	0.74	0.58–0.95	0.018	0.75	0.57–0.98	0.033		0.75	0.58–0.98	0.033
Waist-hip ratio							6th			
Normal	1.00	–-		1.00	–-			1.00	–-	
Abnormal	1.01	0.84–1.23	0.893	1.29	1.03–1.62	0.026		1.33	1.07–1.67	0.011
Education level							–-			
Illiterate	1.00	–-		1.00	–-					
Primary school	0.82	0.67–1.02	0.070	0.88	0.69–1.12	0.299				
Secondary school and above	0.82	0.66–1.03	0.085	0.86	0.66–1.13	0.289				
Lifestyle										
Smoking							–-			
Never	1.00	–-		1.00	–-					
Current smoker	1.87	1.43–2.43	< 0.001	1.36	0.97–1.90	0.079				
Quit	1.91	1.54–2.37	< 0.001	1.21	0.89–1.65	0.225				
Alcohol drinking							–-			
Never	1.00	–-		1.00	–-					
Current drinker	0.90	0.68–1.19	0.474	0.86	0.62–1.17	0.331				
Quit	1.89	1.38–2.59	< 0.001	1.35	0.93–1.95	0.111				

### Subgroup analysis

**Fig. 1:**
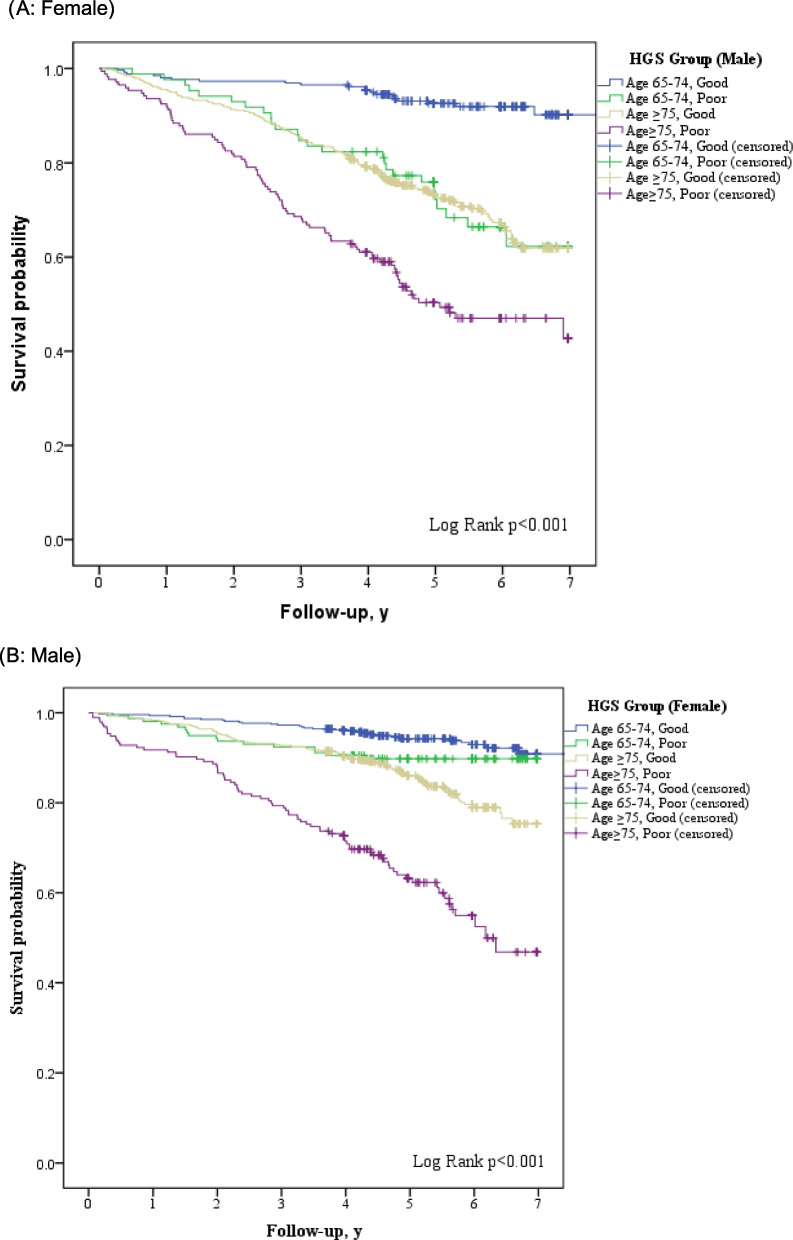
**A** and **B** are survival curves by gender, age and HGS

Table 3 displays the estimated mortality rates among different subgroups. For individuals with good HGS status, the mortality rates per 100 person-years were 1.68 for men and 1.40 for women aged 65–74 years, and 7.29 for men and 3.80 for women aged 75 years or older. In contrast, for individuals with poor HGS status, the mortality rates were significantly higher, with values of 7.08 for men and 2.25 for women aged 65–74 years, and 14.16 for men and 10.53 for women aged 75 years or older. The survival curves over a 7-year follow-up period for the community-dwelling Taiwanese population, stratified by age group and HGS status, are illustrated in Fig. 1A and B for females and males, respectively.

**Table 3:** Mortality rates and adjusted hazard ratios of hand grip strength for death by age and gender

Gender	Age (years)	Mortality rate (1/100 person-year)		Adjusted hazard ratio	95%CI	*P*-value
		Good hand grip strength	Poor hand grip strength			
Male	65–74	1.68	7.08	4.12	2.16–7.84	< 0.001
	≥ 75	7.29	14.16	1.49	1.07–2.07	0.017
Female	65–74	1.40	2.25	1.27	0.65–2.48	0.490
	≥ 75	3.80	10.53	2.09	1.43–3.04	< 0.001

Additionally, stratified Cox regression analyses were performed for men and women in both age groups (65–74 years and 75 years or older). The results demonstrated that older Taiwanese individuals with poor HGS status had significantly higher mortality rates among men aged 65–74 years (adjusted HR 4.12, 95% CI: 2.16–7.84), men aged 75 years or older (adjusted HR 1.49, 95% CI: 1.07–2.07), and women aged 75 years or older (adjusted HR 2.09, 95% CI: 1.43–3.04).

## Discussions

Our study results suggested that poor HGS status independently increased the risk of middle-term all-cause mortality of the community-dwelling older population in Taiwan. Our previous investigations conducted in the Yilan region have also demonstrated an association between lower HGS and various unfavorable health outcomes, including slower walking speed and increased incidence of falls [37, 38]. These findings highlight the importance of considering HGS as a valuable indicator for assessing risk and mortality among older individuals residing in the community.

In the field of community medicine and preventive medicine, our study results also disclosed those community-dwelling older adults with poor HGS had significantly higher mortality rate. This result was compatible with those studies conducted in Asian regions, such as in Singapore (target population were aged 65 years and above) [22], Korea (middle and older adults) [21], and Japan (aged 65–89 years) [7].Similar results had also been noted in non-Asian regions. For instance, a pooled analysis involving 5,250 nondependent community-dwelling older individuals aged 60 years and above in Chile revealed that the lowest quantile of HGS was associated with an increased risk of all-cause mortality [23]. From the results of SHARE study, it was estimated that a 5 kg increase in HGS was associated with a reduced risk of all-cause mortality in adults aged 50 years or older [20]. These findings from diverse populations and regions emphasized the relevance and significance of HGS measurements in assessing mortality risk, and also provided valuable insights into the potential utility of HGS as a predictor of health outcomes. However, even with these efforts, the information about the impacts of HGS on the mortality in more specific age and sex cohorts of the older people was still limited.

After subdividing the older community-dwelling population into more specific age and gender cohorts, our study results disclosed that the risk of death was significantly higher in people aged 75 years and older with poor HGS, and also in men aged 65–74 years with poor HGS. But the difference was not significant in women aged 65–74 years. Though the underlying causes of the differences needed further investigation, we believed that life style might be one of the underlying causes. Because in Yilan, which has been a rural environment and older people have closely followed the tradition, usually women would serve for the household and farming work, and men, except for farming work, would go outside serving for the factory or government work. Therefore, around the age of 65 years, men would face the retirement issues. Several previous studies had suggested that retirement, which might lead to loneliness and social isolation, would have negative impacts on the health outcomes [40, 41].This life style might partly explain the difference of the risk of mortality between men and women aged 65–74 years and needed to be further investigated. But this result also supported our estimation that we need to stratify the older population into more various and specific cohorts in order to provide more appropriate strategies to prolong their life and increase their quality of life (QoL). Besides, the higher mortality rates for men, especially in male with 75 years or older, might be due to shorter life expectancy for men in Taiwan and relatively short following-up period of this study. However, this estimation still needs further observation and investigation.

Our study results also suggested that additional variables, including CVD medical history, BMI, and WHR, were also found to significantly influence 7-year survival, in addition to age, gender, and HGS. Because CVD usually represented the consequence of many other chronic diseases (such as diabetes, hypertension and hyperlipidemia) and bore the possible fatal sequelae, it would not be surprising that CVD would increase the mortality risk. However, it was also interesting that both overweight and obese older population had lower HR’s of mortality as compared to that of the population with normal weight. Several studies have suggested the limitations of BMI as an index of obesity, as so-called the obesity paradox [42, 43]. Han SY et al. also proposed that because of the inability of BMI to distinguish between lean body mass and body fat, which were important in the differentiation of sarcopenia and frailty, and of the important role of abdominal and visceral fat in the pathogenesis of adverse outcomes of many chronic diseases, waist circumference (WC) might be a more appropriate indicator of the risks of myocardial infarction in the older population [44]. In one of our previous studies, we also found that overweight older individuals had better mental health-related quality of life (HRQoL), self-rated health (SRH) and happiness [45]. The results of this study provided evidence that after controlling the impacts of abdominal and visceral fat (represented by WHR), higher level of BMI (in the range of overweight and obesity) might disclose the protective effects (such as the positive influence on prothrombotic factors or the production of certain cytokines) against the risk of mortality [42, 44]. Further investigations are still needed to validate the theory. Our findings also highlighted the multifactorial nature of survival outcomes in older individuals, as the results from the UK Biobank [39].

It has been noted that the poor HGS group had higher proportions of individuals with a non-alcohol drinking lifestyle as compared to the good HGS group. But the association disappeared after the Cox proportional regression analysis. It seemed that there might be some confounding factors and one of them might be the higher proportion of women. The proportion of women with an alcohol drinking lifestyle has been relatively low and some of them might get chronic diseases early in life and therefore be prevented from drinking. This estimation also needed to be further studied.

Furthermore, there is growing evidence suggesting that HGS may serve as a valuable tool for predicting cognitive decline among older adults [46, 47], a factor that has been associated with subsequent mortality [48, 49]. Given this potential link, future studies could explore the predictive capacity of HGS for cognitive decline and its subsequent impact on mortality outcomes. Additionally, further investigations could be designed and conducted based on the data obtained from the Yilan study, allowing for a more comprehensive understanding of the associations between HGS, cognitive decline, and mortality [50].

The present study has several strengths. First, the study conducted a seven-year longitudinal follow-up and included 2,468 community-dwelling older individuals, which allowed for a more comprehensive analysis of the relationship between HGS and mortality. Longitudinal studies provide valuable insights into the long-term effects of variables on outcomes and a large sample size increases the statistical power. Second, the current investigation was conducted by proficiently trained assistants using a standardized protocol. Comprehensive assessments of physical activities or performances were challenging to administers, necessitating the implementation of home visits to reach the participants. Third, this study identified specific subgroups that are more vulnerable to the adverse effects of poor hand grip strength on mortality. It highlighted the increased mortality risk in males aged 65–74 years and females and males aged 75 years or older with poor HGS. This information helps to design target interventions and to allocate resources more effectively. The limitations of this study included limited generalizability of the findings to other populations, potential unknown confounding factors, and the absence of long-term measurement of HGS variation. Further research is necessary to investigate whether similar associations exist in different regions and diverse groups, and to provide more comprehensive evaluations of the impact of HGS on mortality risk. Long-term interventions could be valuable in determining whether improvements in HGS lead to decreased mortality rates among the older individuals.

## Conclusion

The current investigation utilized a longitudinal methodology, tracking a cohort of older adults residing in the community. The findings revealed a significant association between decreased HGS and mortality, indicating an increased risk of death among male individuals aged 65–74 years and both female and male individuals aged 75 years and above. Our study contributes to the growing body of evidence on the role of HGS as a predictor of mortality among the older people, and underscores the needs for further stratification of older people into specific age and gender cohorts for more adequate and suitable management of their health.

### Supplementary Information

**Additional file 1:** **Table S1.** Multivariable Cox proportional hazard models of death.

## Data Availability

The datasets used and/or analyzed during the current study are available from the corresponding author on reasonable request.

## References

[1] GBD 2019 Risk Factors Collaborators (2020). Global burden of 87 risk factors in 204 countries and territories, 1990–2019: a systematic analysis for the Global Burden of Disease Study 2019. Lancet.

[2] Chen LK, Hwang AC, Liu LK, Lee WJ, Peng LN (2016). Frailty is a geriatric syndrome characterized by multiple impairments: a comprehensive approach is needed. J Frailty Aging.

[3] Dent E, Martin FC, Bergman H, Woo J, Romero-Ortuno R, Walston JD (2019). Management of frailty: opportunities, challenges, and future directions. Lancet.

[4] Chen LK, Liu LK, Woo J, Assantachai P, Auyeung TW, Bahyah KS, Chou MY, Chen LY, Hsu PS, Krairit O (2014). Sarcopenia in Asia: consensus report of the Asian Working Group for Sarcopenia. J Am Med Dir Assoc.

[5] Cruz-Jentoft AJ, Bahat G, Bauer J, Boirie Y, Bruyère O, Cederholm T, Cooper C, Landi F, Rolland Y, Sayer AA (2019). Sarcopenia: revised European consensus on definition and diagnosis. Age Ageing.

[6] Chainani V, Shaharyar S, Dave K, Choksi V, Ravindranathan S, Hanno R, Jamal O, Abdo A, Abi Rafeh N (2016). Objective measures of the frailty syndrome (hand grip strength and gait speed) and cardiovascular mortality: a systematic review. Int J Cardiol.

[7] Nofuji Y, Shinkai S, Taniguchi Y, Amano H, Nishi M, Murayama H, Fujiwara Y, Suzuki T (2016). Associations of walking speed, grip strength, and standing balance with total and cause-specific mortality in a general population of Japanese elders. J Am Med Dir Assoc.

[8] Yates T, Zaccardi F, Dhalwani NN, Davies MJ, Bakrania K, Celis-Morales CA, Gill JMR, Franks PW, Khunti K (2017). Association of walking pace and handgrip strength with all-cause, cardiovascular, and cancer mortality: a UK Biobank observational study. Eur Heart J.

[9] Prasitsiriphon O, Pothisiri W (2018). Associations of grip strength and change in grip strength with all-cause and cardiovascular mortality in a European older population. Clin Med Insights Cardiol.

[10] Kim GR, Sun J, Han M, Park S, Nam CM (2019). Impact of handgrip strength on cardiovascular, cancer and all-cause mortality in the Korean longitudinal study of ageing. BMJ Open.

[11] Contreras-Bolívar V, Sánchez-Torralvo FJ, Ruiz-Vico M, González-Almendros I, Barrios M, Padín S, Alba E, Olveira G (2019). GLIM criteria using hand grip strength adequately predict six-month mortality in cancer inpatients. Nutrients.

[12] Zhuang CL, Zhang FM, Li W, Wang KH, Xu HX, Song CH, Guo ZQ, Shi HP (2020). Associations of low handgrip strength with cancer mortality: a multicentre observational study. J Cachexia Sarcopenia Muscle.

[13] Song M, Zhang Q, Tang M, Zhang X, Ruan G, Zhang X, Zhang K, Ge Y, Yang M, Li Q (2021). Associations of low hand grip strength with 1 year mortality of cancer cachexia: a multicentre observational study. J Cachexia Sarcopenia Muscle.

[14] Xu BY, Yan S, Low LL, Vasanwala FF, Low SG (2019). Predictors of poor functional outcomes and mortality in patients with hip fracture: a systematic review. BMC Musculoskelet Disord.

[15] Gutiérrez-Hermosillo H, de León-González ED, Medina-Chávez JH, Torres-Naranjo F, Martínez-Cordero C, Ferrari S (2020). Hand grip strength and early mortality after hip fracture. Arch Osteoporos.

[16] Alajlouni DA, Bliuc D, Tran TS, Blank RD, Cawthon PM, Ensrud KE, Lane NE, Orwoll ES, Cauley JA, Center JR (2022). Muscle strength and physical performance are associated with risk of postfracture mortality but not subsequent fracture in men. J Bone Miner Res.

[17] Charatcharoenwitthaya P, Karaketklang K, Aekplakorn W (2022). Muscle strength, but not body mass index, is associated with mortality in patients with non-alcoholic fatty liver disease. J Cachexia Sarcopenia Muscle.

[18] Hamasaki H (2021). What can hand grip strength tell us about type 2 diabetes?: mortality, morbidities and risk of diabetes. Expert Rev Endocrinol Metab.

[19] Dai KZ, Laber EB, Chen H, Mentz RJ, Malhotra C (2023). Hand grip strength predicts mortality and quality of life in heart failure: insights from the Singapore cohort of patients with advanced heart failure. J Card Fail.

[20] López-Bueno R, Andersen LL, Calatayud J, Casaña J, Smith L, Jacob L, Koyanagi A, López-Gil JF, Del Pozo CB (2022). Longitudinal association of handgrip strength with all-cause and cardiovascular mortality in older adults using a causal framework. Exp Gerontol.

[21] Kim J (2021). Handgrip strength to predict the risk of all-cause and premature mortality in Korean adults: a 10-year cohort study. Int J Environ Res Public Health.

[22] Malhotra R, Tareque MI, Tan NC, Ma S (2020). Association of baseline hand grip strength and annual change in hand grip strength with mortality among older people. Arch Gerontol Geriatr.

[23] Lera L, Albala C, Leyton B, Márquez C, Angel B, Saguez R, Sánchez H (2018). Reference values of hand-grip dynamometry and the relationship between low strength and mortality in older Chileans. Clin Interv Aging.

[24] Wang Y, Liu Y, Hu J, Guan H, Wang Y, Liu M, He L, Sun N, Yang W, Ma Y (2022). Association of handgrip strength with all-cause mortality: a nationally longitudinal cohort study in China. J Sci Med Sport.

[25] Xie K, Lu Z, Han X, Huang M, Wang J, Kou S, Wang W, Zhuang S, Zheng W (2022). Handgrip strength as an indicator for death events in China: A longitudinal cohort study. PLoS ONE.

[26] Cai Y, Liu L, Wang J, Gao Y, Guo Z, Ping Z (2021). Linear association between grip strength and all-cause mortality among the elderly: results from the SHARE study. Aging Clin Exp Res.

[27] López-Bueno R, Andersen LL, Calatayud J, Casaña J, Grabovac I, Oberndorfer M, Del Pozo Cruz B (2022). Associations of handgrip strength with all-cause and cancer mortality in older adults: a prospective cohort study in 28 countries. Age Ageing.

[28] Wu Y, Wang W, Liu T, Zhang D (2017). Association of grip strength with risk of all-cause mortality, cardiovascular diseases, and cancer in community-dwelling populations: a meta-analysis of prospective cohort studies. J Am Med Dir Assoc.

[29] Núñez-Cortés R, Cruz BDP, Gallardo-Gómez D, Calatayud J, Cruz-Montecinos C, López-Gil JF, López-Bueno R (2022). Handgrip strength measurement protocols for all-cause and cause-specific mortality outcomes in more than 3 million participants: A systematic review and meta-regression analysis. Clin Nutr.

[30] López-Bueno R, Andersen LL, Koyanagi A, Núñez-Cortés R, Calatayud J, Casaña J, Del Pozo CB (2022). Thresholds of handgrip strength for all-cause, cancer, and cardiovascular mortality: a systematic review with dose-response meta-analysis. Ageing Res Rev.

[31] Gómez-Campos R, Vidal Espinoza R, De Arruda M, Ronque ER, Urra-Albornoz C, Minango JC, Alvear-Vasquez F, la Torre Choque CD, Castelli Correia de Campos LF, Sulla Torres J (2022). Relationship between age and handgrip strength: Proposal of reference values from infancy to senescence. Front Public Health.

[32] Riviati N, Setiati S, Laksmi PW, Abdullah M (2017). Factors related with handgrip strength in elderly patients. Acta Med Indones.

[33] Rosas-Carrasco O, Núñez-Fritsche G, López-Teros MT, Acosta-Méndez P, Cruz-Oñate JC, Navarrete-Cendejas AY, Delgado-Moreno G (2022). Low muscle strength and low phase angle predicts greater risk to mortality than severity scales (APACHE, SOFA, and CURB-65) in adults hospitalized for SARS-CoV-2 pneumonia. Front Nutr.

[34] Zhang XM, Jiao J, Zhu C, Guo N, Liu Y, Lv D, Wang H, Jin J, Wen X, Zhao S (2021). Association between low handgrip strength and 90-day mortality among older Chinese inpatients: a national multicenter prospective cohort study. Front Nutr.

[35] Pan PJ, Lin CH, Yang NP, Chen HC, Tsao HM, Chou P, Hsu NW (2020). Normative data and associated factors of hand grip strength among elderly individuals: the Yilan study. Taiwan Sci Rep.

[36] Chen HC, Hsu NW, Chou P (2017). The association between sleep duration and hand grip strength in community-dwelling older adults: the Yilan study Taiwan. Sleep.

[37] Yang NP, Hsu NW, Lin CH, Chen HC, Tsao HM, Lo SS, Chou P (2018). Relationship between muscle strength and fall episodes among the elderly: the Yilan study. Taiwan BMC Geriatr.

[38] Lin YH, Chen HC, Hsu NW, Chou P (2021). Using hand grip strength to detect slow walking speed in older adults: the Yilan study. BMC Geriatr.

[39] Zaccardi F, Franks PW, Dudbridge F, Davies MJ, Khunti K, Yates T (2021). Mortality risk comparing walking pace to handgrip strength and a healthy lifestyle: a UK biobank study. Eur J Prev Cardiol.

[40] Hong JH, Nakamura JS, Berkman LF, Chen FS, Shiba K, Chen Y, Kim ES, VanderWeele TJ (2023). Are loneliness and social isolation equal threats to health and well-being? An outcome-wide longitudinal approach. SSM - Population Health.

[41] Xu Y (2023). The effect of retirement on health and mortality in the United States. J Popul Res.

[42] Dramé M, Godaert L (2023). The obesity paradox and mortality in older adults: a systematic review. Nutrients.

[43] Wang L, Yi Z (2022). Obesity paradox and aging: Visceral Adiposity Index and all-cause mortality in older individuals: a prospective cohort study. Front Endocrinol (Lausanne).

[44] Han SY, Kim NH, Kim DH, Kim YH, Park YK, Kim SM (2022). Associations between body mass index, waist circumference, and myocardial infarction in older adults aged over 75 years: a population-based cohort study. Medicina (Kaunas).

[45] Chang HT, Hsu NW, Chen HC, Tsao HM, Lo SS, Chou P (2018). Associations between body mass index and subjective health outcomes among older adults: findings from the Yilan study Taiwan. Int J Environ Res Public Health.

[46] Chou MY, Nishita Y, Nakagawa T, Tange C, Tomida M, Shimokata H, Otsuka R, Chen LK, Arai H (2019). Role of gait speed and grip strength in predicting 10-year cognitive decline among community-dwelling older people. BMC Geriatr.

[47] Luo J, Su L, Ndeke JM, Wang F, Hendryx M (2022). Gait speed, handgrip strength, and cognitive impairment among older women - a multistate analysis. Exp Gerontol.

[48] Su Y, Dong J, Sun J, Zhang Y, Ma S, Li M, Zhang A, Cheng B, Cai S, Bao Q (2021). Cognitive function assessed by Mini-mental state examination and risk of all-cause mortality: a community-based prospective cohort study. BMC Geriatr.

[49] Li Z, Gong X, Wang S, Liu M, Liu S, Wang Y, Wu D, Yang M, Li R, Li H (2022). Cognitive impairment assessed by mini-mental state examination predicts all-cause and CVD mortality in Chinese older adults: a 10-year follow-up study. Front Public Health.

[50] Ang SH, Hsu NW, Tsai PH, Pan PJ, Chen HC, Chou P, Lin KC (2022). Different item characteristics of a mild cognitive impairment screening tool in the community-based Yilan Study: application of the item response theory. Psychogeriatrics.

