# Supplementary material for: Handgrip strength is associated with mortality in community-dwelling older adults: the Yilan cohort study, Taiwan
Source: BMC Public Health. 2023 Nov 8;23:2194. doi: 10.1186/s12889-023-17058-9 (PMC10631044; doi:10.1186/s12889-023-17058-9)
Supplement: Supplementary file 1 — Additional file 1: Table S1. Multivariable Cox proportional hazard models of death. [file 12889_2023_17058_MOESM1_ESM.docx]

**Table S1. Multivariable Cox proportional hazard models of death**

| Variables | Hazard ratio | 95%CI | P-value |  | Step | Hazard ratio | 95%CI | P-value |
| --- | --- | --- | --- | --- | --- | --- | --- | --- |
| Age (years) |  |  |  |  | 1st |  |  |  |
| 65-74 | 1.00 | --- |  |  |  | 1.00 | --- |  |
| ≥75 | 2.09 | 1.63-2.69 | <0.001 |  |  | 2.13 | 1.66-2.72 | <0.001 |
| Gender |  |  |  |  | 2rd |  |  |  |
| Female | 1.00 | --- |  |  |  | 1.00 | --- |  |
| Male | 2.63 | 1.95-3.54 | <0.001 |  |  | 3.10 | 2.45-3.92 | <0.001 |
| Hand grip strength | 0.95 | 0.94-0.97 | <0.001 |  | 3nd | 0.95 | 0.94-0.97 | <0.001 |
| Medical history |  |  |  |  |  |  |  |  |
| Cardiovascular diseases | 1.61 | 1.32-1.98 | <0.001 |  | 4th | 1.66 | 1.36-2.01 | <0.001 |
| Hypertension | 1.12 | 0.90-1.39 | 0.324 |  | --- |  |  |  |
| Diabetes | 0.99 | 0.78-1.25 | 0.908 |  | --- |  |  |  |
| Hyperlipidemia | 0.94 | 0.73-1.20 | 0.610 |  | --- |  |  |  |
| BMI (kg/m^2^) |  |  |  |  | 5th |  |  |  |
| <18.5 | 1.68 | 1.15-2.46 | 0.007 |  |  | 1.66 | 1.15-2.41 | 0.007 |
| 18.5–23.9 | 1.00 | --- |  |  |  | 1.00 | --- |  |
| 24–26.9 | 0.73 | 0.56-0.93 | 0.012 |  |  | 0.72 | 0.56-0.92 | 0.009 |
| ≥27 | 0.77 | 0.59-1.00 | 0.054 |  |  | 0.77 | 0.59-1.00 | 0.052 |
| Waist-hip ratio |  |  |  |  | 6th |  |  |  |
| Normal | 1.00 | --- |  |  |  | 1.00 | --- |  |
| Abnormal | 1.27 | 1.02-1.59 | 0.036 |  |  | 1.31 | 1.05-1.63 | 0.018 |
| Education level |  |  |  |  | --- |  |  |  |
| Illiterate | 1.00 | --- |  |  |  |  |  |  |
| Primary school | 0.90 | 0.71-1.15 | 0.408 |  |  |  |  |  |
| Secondary school and above | 0.90 | 0.69-1.18 | 0.445 |  |  |  |  |  |
| Lifestyle |  |  |  |  |  |  |  |  |
| Smoking |  |  |  |  | --- |  |  |  |
| Never | 1.00 | --- |  |  |  |  |  |  |
| Current smoker | 1.39 | 0.99-1.95 | 0.055 |  |  |  |  |  |
| Quit | 1.22 | 0.90-1.66 | 0.204 |  |  |  |  |  |
| Alcohol drinking |  |  |  |  | --- |  |  |  |
| Never | 1.00 | --- |  |  |  |  |  |  |
| Current drinker | 0.90 | 0.66-1.23 | 0.509 |  |  |  |  |  |
| Quit | 1.33 | 0.92-1.92 | 0.130 |  |  |  |  |  |
